# Effect of breastfeeding on cardiometabolic risk in patients with polycystic ovary syndrome

**DOI:** 10.1007/s40618-026-02948-7

**Published:** 2026-06-22

**Authors:** Stefano Palomba, Giuseppe Seminara, Flavia Costanzi, Rossella Cannarella, Tiziana Russo, Fabrizio Signore, Martina Sarica, Aldo E. Calogero, Donatella Caserta, Antonio Aversa

**Affiliations:** 1 Department of Medical-Surgical Sciences and Translational Medicine, University Sapienza of Rome, Rome, 00185 Italy; 2 Unit of Obstetrics and Gynecology, Grande Ospedale Metropolitano of Reggio Calabria, Reggio Calabria, 89124 Italy; 3 Department of Experimental and Clinical Medicine, University “Magna Graecia” of Catanzaro, Catanzaro, 88100 Italy; 4 Endocrinology Unit, “Renato Dulbecco” University Hospital, Catanzaro, 88100 Italy; 5 Department of Clinical and Experimental Medicine, University of Catania, Catania, 95124 Italy; 6 Obstetrics and Gynaecology Department, USL Roma 2, Sant’Eugenio Hospital, Rome, 00144 Italy; 7 Unit of Gynecology, Sant’Andrea Hospital, Rome, 00189 Italy

**Keywords:** Breastfeeding, PCOS, Polycystic ovary syndrome, Gestational diabetes, Hypertension, Pregnancy complications

## Abstract

**Purpose:**

This study investigated whether breastfeeding (BF) is associated with a reduction in persistent hypertension (PH) and glucose metabolism impairment in patients with polycystic ovary syndrome (PCOS) who experienced pregnancy-induced hypertension (PIH) and/or gestational diabetes mellitus (GDM).

**Methods:**

This secondary observational analysis utilized data from two prospective cohort studies involving 241 participants (101 PCOS patients; 140 controls) with a history of GDM and/or PIH. At 18 months post-delivery, patients underwent physical examinations, blood pressure monitoring, and biochemical assays, including oral glucose tolerance tests. Feeding practices were categorized as exclusive BF, partial BF, or formula feeding (FF).

**Results:**

Patients with PCOS demonstrated significantly shorter BF durations (9.6 ± 7.0 vs. 12.6 ± 5.9 months; *P* < 0.001) and lower exclusive BF rates (39.6% vs. 62.9%; *P* < 0.001) compared to controls. At 18 months, the composite cardiometabolic outcome prevalence was 55.4% in the PCOS group versus 11.4% in controls (*P* < 0.001). Multivariate analysis identified exclusive BF as a potent independent factor associated with a significantly lower risk (adjusted OR 0.14, 95% CI 0.05–0.38; *P* < 0.001) of cardiometabolic impairment.

**Conclusion:**

Breastfeeding is associated with a significant reduction in long-term cardiometabolic risks in women with PCOS following complicated pregnancies. Supporting and optimizing lactation may represent a valuable non-pharmacological strategy to consider for this high-risk population.

**Supplementary Information:**

The online version contains supplementary material available at https://doi.org/10.1007/s40618-026-02948-7.

## Introduction

Breastfeeding (BF) is recognized as a reliable method to promote the health of both mothers and infants [1]. Infants who are breastfed for extended periods tend to experience fewer infections and lower mortality rates, reduced risk of dental misalignment, and enhanced cognitive development compared to those breastfed for shorter durations or not at all [1, 2]. Additionally, BF has been associated with lower risks of breast cancer, better birth spacing, and a reduced risk of developing type 2 diabetes (DM2) and ovarian cancer for mothers [1]. Consequently, leading health authorities recommend exclusive BF at least for the first six months of life [3].

The protective effects of BF include a significantly reduced cardiometabolic risk after pregnancy, especially in those with gestational diabetes mellitus (GDM) [4, 5]. Moreover, the intensity and duration of BF are positively associated with slower infant growth curves compared with formula feeding (FF), indicating the positive effect of BF on growth trends in contrast to the increased risk of obesity and metabolic diseases observed in infants exposed to GDM [5].

Polycystic ovary syndrome (PCOS) is among the most prevalent endocrine disorders, affecting a significant proportion of women of reproductive age [6]. Women with PCOS are more susceptible to pregnancy-related complications, including both maternal and neonatal outcomes [7]. The main maternal complications associated with PCOS include GDM and pregnancy-induced hypertension (PIH)/preeclampsia (PE), with estimated prevalence rates of 13% and 5–7%, respectively [8]. However, how different PCOS phenotypes influence these complications remains uncertain [7–9]. Furthermore, large-scale meta-analyses have confirmed that women with PCOS are at elevated long-term risk for cardiometabolic conditions, such as hypertension and DM2 [10].

In the general population, strong associations have been observed between GDM, PIH, and the later development or persistence of cardiovascular disease (CVD) [11]. Our earlier studies showed that women with PCOS face a significantly higher risk of persistent abnormalities in glucose metabolism and elevated blood pressure following GDM and PIH/PE, compared to women without PCOS [12–14]. Specifically, PCOS patients had more than a threefold higher risk of developing conditions such as impaired glucose tolerance (IGT), impaired fasting glucose (IFG), and DM2 18 months after childbirth, relative to age- and body mass index (BMI)-matched controls [12]. In patients with PCOS who had a history of PIH, the chances of developing persistent hypertension (PH) were 5, 4, and 6 times higher than those of BMI-matched non-PCOS controls at 6, 12, and 18 months after delivery, respectively [13, 14].

One of the mechanisms potentially involved in the higher prevalence of persistent abnormalities in glucose metabolism and elevated blood pressure in patients with PCOS and history of GDM and/or PIH includes abnormalities in BF. Moreover, there is limited research examining BF in PCOS now [15–18]. However, considering the known benefits of BF in enhancing insulin sensitivity [19], it is plausible that lactation optimization could impact the increased cardiometabolic risk seen in PCOS patients who experience pregnancy complications [4, 5]. Based on these considerations, we designed the current study to test the hypothesis that BF is associated with a reduction of the PH and/or glucose metabolism impairment in patients with PCOS who experienced pregnancy complications, i.e. GDM and/or PIH/PE, playing a potential role as preventive intervention for lowering cardiometabolic risk in this high-risk population.

## Materials and methods

### Study population

This is a secondary observational analysis based on the two prospective cohort studies previously published by our group and conducted in two different Italian general hospitals [12–14]. The original studies were designed to evaluate cardiometabolic outcomes in women with and without PCOS who experienced GDM [12] and PIH/PE at short- [13] and long-term [14] follow-up.

The primary objective was to evaluate, in overall population and in each cohort, the association between BF and long-term cardiometabolic outcomes, i.e. after 18 months from delivery, in patients with and without PCOS. Specifically, data prospectively recorded from two cohorts [12, 14] were re-analyzed in a retrospective fashion to assess the influence of BF on patients with and without PCOS after high-risk pregnancies, on the PH and/or glucose metabolism impairment, respectively. The diagnostic criteria of these conditions were aligned according to the most recent guidelines [20–23].

PCOS diagnosis was made in a retrospective fashion applying contemporary national and international criteria [24, 25] to clinical data derived from patient history and clinical reports. Specifically, PCOS diagnosis were performed when two of the three following criteria were satisfied, i.e. irregular menstrual cycles (cycle length less than 21 or more than 35 days, or less than 8 cycles per year), signs of clinical (modified Ferriman Gallwey score of 6) and/or biochemical hyperandrogenism (free androgen index higher than 6), and evidence of polycystic ovary morphology (PCOM) on transvaginal ultrasonography using transducer band-width frequency > 8 MHz (follicle number per ovary ≥ 20 in at least 1 ovary and/or an ovary volume > 10 ml) [24, 25]. On the other hand, the same diagnostic criteria were excluded in control group.

The exclusion criteria for all patients included incomplete or unclear data regarding PCOS diagnosis or exclusion, chronic hypertension (preexisting hypertension before pregnancy or before 20 weeks’ gestation), secondary hypertension, superimposed PE (development of PE in patients with preexisting hypertension), or preexisting DM or impaired glucose tolerance. Other exclusion criteria were age of < 18 or > 38 years, a gestational age at the GDM diagnosis that was > 28 or < 24 weeks, drug or alcohol abuse, cigarette smoking, multiple pregnancies, concurrent medical conditions (e.g., antiphospholipid antibody syndrome, chronic kidney disease, systemic lupus, and autoimmune disease), and patients on aspirin, calcium supplements, antidiabetic drugs (e.g., metformin), and medications affecting SBP/DBP. No participants were excluded for the specific purposes of this secondary analysis; thus, the study population consists entirely of the ‘completers’ from primary studies.

### Study protocol

At the beginning of the study and throughout pregnancy, each patient was closely monitored with clinical, biochemical and ultrasonographic assessments according to a predefined schedule. Data on socioeconomic status, work and educational background, associated medical conditions, and family history were collected for each participant.

Clinical management during and after pregnancy followed common clinical practice. Specifically, clinical and ultrasonographic assessments were performed at study entry and every 4 weeks until delivery in patients with PIH and GDM or every 2 weeks in patients with PE without FGR. Clinical evaluations included obstetric examination, anthropometric measurements, heart rate, and systolic blood pressure (SBP)/diastolic BP (DBP) assessments. Body weight changes during pregnancy were also monitored and recorded.

Dietary recommendations were individualized based on pre-pregnancy BMI and metabolic status, in accordance with standard clinical guidelines for the management of GDM and hypertensive disorders of pregnancy. In particular, hypocaloric dietary regimens were prescribed only in overweight or obese patients, while normocaloric balanced diets were recommended for normal-weight women. Sodium restriction was suggested in patients with hypertensive disorders according to guideline-based clinical practice. The physical activity program consisted mainly of moderate-intensity aerobic exercise (e.g., walking), performed at least 3 times per week for approximately 30 min per session. Adherence to diet and exercise protocols was evaluated at each visit.

All data were assessed in all patients after 18 months from delivery. Lifestyle recommendations, including dietary advice and physical activity, were continued after delivery according to the same individualized approach. At each time-point, participants underwent physical examination and blood pressure measurement using standardized protocols. Glucose metabolism was assessed through fasting plasma glucose and insulin levels. Oral glucose tolerance test (OGTT) was executed between 4 and 12 weeks after delivery in patients previously affected by GDM [26] and subsequently repeated at 18 months in patients who had not already met the criteria for a diagnosis of DM2. Cardiometabolic impairment was defined as glucose metabolism alterations or PH. Alterations in glucose metabolism were defined as IFG (plasma fasting glucose ≥ 100 mg/dL and ≤ 125 mg/dL), IGT (plasma glucose ≥ 140 mg/dL and ≤ 199 mg/dL 2 h after a 75-g OGTT) or DM2 (twice plasma fasting glucose ≥ 126 mg/dL, plasma glucose ≥ 200 mg/dL 2 h after a 75-g OGTT, HbA1c ≥ 48 mmol/mol or random plasma glucose ≥ 200 mg/dL in presence of classic symptoms of hyperglycemia or hyperglycemic crisis) [20]. PH was defined as SBP ≥ 130 mmHg and/or DBP ≥ 85 mmHg [21].

A composite cardiometabolic outcome was also defined, including the presence of at least one of the following four conditions at 18-month follow-up visit after delivery: PH, IFG, IGT, or DM2. This composite endpoint was used to capture the overall cardiometabolic risk profile of the study population and to increase the statistical study power.

The original datasets included detailed information on BF, including both its duration and exclusivity. These data were collected during scheduled follow-up visits post-delivery. At each visit, a structured and standardized interview was conducted by trained medical personnel to determine the infant’s feeding status. To ensure accuracy and reduce recall bias, participants were asked specific questions regarding the introduction of water, other liquids, or solids. The duration of BF was assessed up to the end of the follow-up period, while exclusivity was determined based on the first 6 months of life. Participants were categorized into exclusive BF, partial BF, or FF, in accordance with definitions recommended by the World Health Organization (WHO) and the United Nations Children’s Fund (UNICEF) [27]. Exclusive BF was defined when the infant received only breast milk until 6 months of age, either directly from the breast or expressed, with no addition of other liquids or solids. The only exceptions permitted were oral rehydration solutions, drops or syrups containing vitamins, minerals, or medicines [27]. Partial BF was defined when the infant received breast milk along with other foods or liquids, including infant formula and/or complementary foods, during the first 6 months of life. FF was defined when the infant received only commercial infant formula, with no intake of breast milk. FF was considered a substitute feeding method in cases where BF was not initiated, was discontinued early, or was not chosen.

### Study design

Data were analyzed in PCOS and non-PCOS cohorts. In particular, the duration of BF during the first 6 months was studied in all patients and compared between patients with and without PCOS diagnosis. At 18-month follow-up visit after delivery, the prevalence of the primary composite cardiometabolic outcomewas analyzed across patients that had exclusive BF, partial BF or FF. A sub-analysis according to individual complications (PH vs. glucose impairment) was also performed to explore specific clinical trajectories.

### Ethics

The original cohort studies were conducted and reported in accordance with the Strengthening the Reporting of Observational Studies in Epidemiology guidelines [28] for observational study reporting. All procedures were in accordance with the Helsinki Declaration on human experimentation. The study protocols were approved by the Institutional Review Boards of the universities of Catanzaro [12] and Sapienza of Rome [13, 14], and thoroughly explained to all participants, and written informed consent was obtained before their inclusion. The present study is a secondary analysis of de-identified data previously collected for those studies, with no new data collection or direct patient contact involved. Additional ethical approval was not required for this type of secondary analysis involving anonymized datasets, as the research poses no additional risk to participants and complies with data protection standards.

### Statistical analysis

Descriptive statistics were utilized to summarize baseline demographic and clinical characteristics, which were presented as means with standard deviations (SD) for continuous variables or as frequencies with percentages for categorical variables. Group comparisons between women with PCOS and non-PCOS controls were performed using the Student’s t-test or Mann-Whitney U test for continuous data, while the chi-square or Fisher’s exact test was applied for categorical variables as appropriate.

To assess the statistical robustness of the primary analysis, a post-hoc power analysis was performed for the composite cardiometabolic outcome within the PCOS subgroups, comparing women who reported exclusive BF with those who reported partial BF or FF. The analysis was based on the observed event rates in the two groups and was conducted using a two-sided significance level (α) of 0.05. Effect size was estimated using Cohen’s h, and the corresponding statistical power was calculated. Fisher’s exact test was used to assess group differences, and the crude odds ratio (OR) was also derived. Conversely, due to the low event rates within individual sub-categories (such as DM2), these individual component analyses were severely underpowered; therefore, they must be considered strictly exploratory and hypothesis-generating only.

To investigate the association between BF practice (as independent risk/protective factor), including both mode and duration, and the risk of glucose abnormalities and PH, multivariable logistic regression models were constructed adjusting data for significant potential confounders, such as maternal age, PCOS status, pregnancy complications including occurrence and severity of GDM and PIH during pregnancy, and BMI changes. Other variables collected at baseline and during follow-up (i.e., educational level, postpartum physical activity, and dietary adherence), were excluded from the final multivariable models based on pre-specified parsimony criteria. Given the number of composite events (*n* = 72), limiting the number of covariates was mandatory to satisfy the events-per-variable guidelines and prevent model overfitting. Furthermore, because dietary adherence and physical activity are strictly collinear with and act as upstream determinants of BMI changes, they were omitted to avoid over-adjustment bias and multicollinearity. In addition, BF effect was dichotomized into exclusive BF vs. non-exclusive BF (partial BF and FF). A further sensitivity analysis was conducted to address the potential for reverse causation and to evaluate the robustness of the observed associations. This analysis involved the construction of an additional multivariable logistic regression model that specifically accounted for the baseline profile of the participants. In that model data were adjusted for pre-pregnancy BMI and PCOS status, in order to determine if the protective effect of exclusive BF remained independent of these initial pre-pregnancy clinical stressors. The results of these associations were also expressed as OR and adjusted OR (aOR) with 95% confidence intervals (CI).

To assess the appropriateness of pooling data from the two original cohorts, a stratified analysis was performed by cohort origin. Furthermore, homogeneity of the treatment effect was tested by including an interaction term (cohort × BF practice) in the multivariable logistic regression models. A non-significant interaction *P*-value was used to confirm that the association between BF (BF duration and exclusive BF) and cardiometabolic outcomes was consistent across both high-risk pregnancy types.

All data were analyzed using MedCalc Software (version 23.0.8, MedCalc Software Ltd, Ostend, Belgium) and IBM SPSS Statistics (version 27, IBM Corp., Armonk, New York, US). The statistical significance was set at a *P* value of less than 0.05 for all analyses performed.

## Results

A total of 241 PCOS patients and non-PCOS controls were included in the study. One hundred twenty-six patients with GDM were enrolled in the first cohort [12] and 115 patients with PHD in the second [14]. General characteristics of population at baseline are shown in Table 1. Considering the whole cohort, 101 patients (41.9%) were identified as PCOS, while 140 (58.1%) were non-PCOS. In the composite cohort, 109 women (45.2%) were diagnosed with GDM, 100 (41.5%) with PIH, and 32 (13.3%) presented both complications.

**Table 1:** General characteristics of the study population at baseline

Parameter	PCOS (*n*. 101)	Controls (*n*. 140)	*P* value
Maternal age (years)	28.7 ± 5.9	28.4 ± 5.8	0.686
Pre-pregnancy weight (kg)	78.0 ± 11.0	73.8 ± 13.4	**0.009**
Height (cm)	165.2 ± 8.9	169.4 ± 8.4	**< 0.001**
Pre-pregnancy BMI (kg/m^2^)	28.2 ± 3.7	27.2 ± 4.6	0.057
Nulliparity (n, %)	72, 71.3	101, 72.1	0.999
Systolic blood pressure (mmHg)	123.2 ± 9.8	119.8 ± 8.4	**0.005**
Diastolic blood pressure (mmHg)	75.9 ± 6.2	76.4 ± 5.1	0.512
GDM (n, %)	54, 53.5	87, 62.1	0.224
PIH (n, %)	69, 68.3	63, 45	**0.001**
Spontaneous pregnancy (n, %)	56, 55.4	110, 78.6	**< 0.001**
Gestation weight gain (kg)	14.8 ± 3.4	14.2 ± 3.1	0.116

Data are expressed as mean ± standard deviations for continuous variables and as number (percentage) for categorical variables. BMI: body mass index; GDM: gestational diabetes mellitus; PIH: pregnancy induced hypertension

The characteristics of the patients at the 18-month follow-up visit are detailed in Table 2. Specifically, 169 women (70.1%) were considered healthy, while 72 (29.9%) presented at least one cardiometabolic alteration. Fifty-one patients had a single cardiometabolic alteration consisting of DM2 (4 patients, 1.6%), IFG (25 patients, 10.4%), IGT (8 patients, 3.3%) or PH (14 patients, 5.8%), while 21 patients had a combination of these alterations (PH + IFG: 8 patients, 3.3%; hypertension + DM2: 3 patients, 1.2%; IFG + IGT: 7 patients, 2.9%; PH + IFG + IGT: 3 patients, 1.2%).

**Table 2:** Cardiometabolic outcomes in patients with and without PCOS and history of PIH and/or GDM after 18 months from delivery

Parameter	PCOS (*n*. 101)	Non-PCOS (*n*. 140)	*P* value
PH (n, %)	23 (22.8)	5 (3.6)	**< 0.001**
DM2 (n, %)	6 (5.9)	1 (0.7)	**0.023**
IGT (n, %)	14 (13.9)	4 (2.9)	**0.002**
IFG (n, %)	34 (33.7)	10 (7.1)	**< 0.001**
Any glycemic alteration * (n, %)	45 (44.6%)	13 (9.3%)	**< 0.001**
Composite outcome ** (n, %)	56 (55.4%)	16 (11.4%)	**< 0.001**
Weight loss (kg)	12.19 ± 4.50	11.46 ± 4.68	0.230
Fasting plasma glucose (mg/dl)	98.19 ± 6.04	82.48 ± 6.15	**< 0.001**
HOMA-IR	3.71 ± 1.43	1.19 ± 0.62	**< 0.001**
HbA1c (mmol/mol)	38.24 ± 5.51	34.77 ± 3.96	**< 0.001**
BMI (kg/m^2^)	27.58 ± 4.87	27.05 ± 3.99	0.354
WC (cm)	106.92 ± 19.43	95.37 ± 16.37	**< 0.001**
Total cholesterol (mg/dL)	195.37 ± 6.11	176.76 ± 5.91	**< 0.001**
HDL cholesterol (mg/dL)	58.70 ± 3.20	68.58 ± 3.38	**< 0.001**
LDL cholesterol (mg/dL)	113.52 ± 7.40	92.99 ± 6.82	**< 0.001**
Triglycerides (mg/dL)	133.10 ± 7.14	99.41 ± 9.43	**< 0.001**

Data are expressed as mean ± standard deviations for continuous variables and as number (percentage) for categorical variables. BMI: body mass index; DM2: type 2 diabetes mellitus; HbA1c: glycated hemoglobin; HOMA-IR: homeostasis model assessment – insulin resistance; IFG: impaired fasting glucose; IGT: impaired glucose tolerance; PCOS: polycystic ovary syndrome; PH: persistent hypertension; WC: waist circumference. * IFG, IGT or DM2; **: IFG, IGT, DM2 or PH

**Fig. 1:**
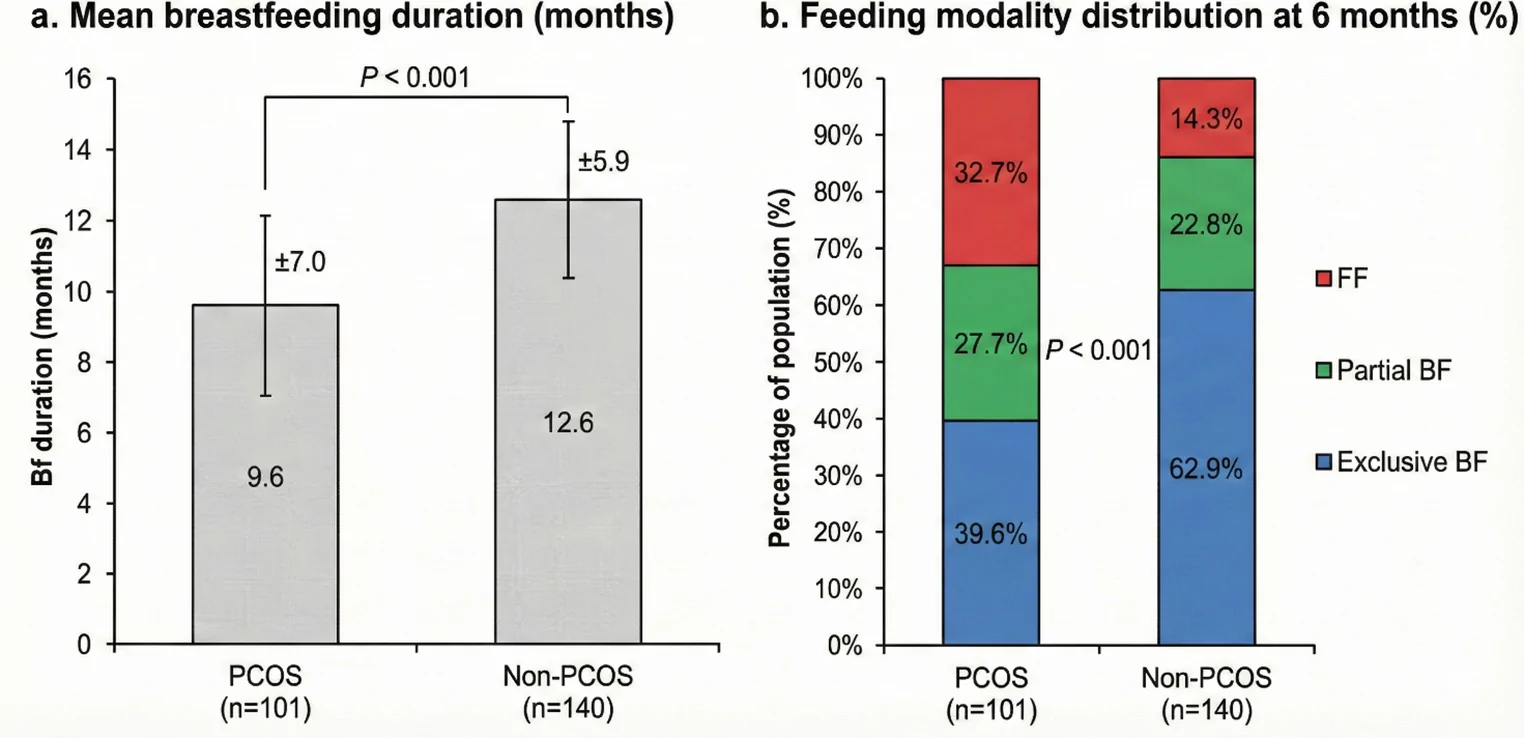
Comparison of breastfeeding (BF) characteristics between women with polycystic ovary syndrome (PCOS) and non-PCOS controls. (**a**) Mean BF duration in months (mean ± SD). (**b**) Distribution of feeding modalities at 18 months after delivery: exclusive BF, partial BF, and formula feeding (FF)

Patients with PCOS showed a significantly higher prevalence of composite cardiometabolic risk when compared to subjects without PCOS (55.4% vs. 11.4%, respectively; *P* < 0.001). Specifically, PH (22.8% vs. 3.6%, *P* < 0.001), DM2 (5.9% vs. 0.7%, *P* = 0.046), any glycemic alterations (44.6% vs. 9.3%, *P* < 0.001) resulted significantly higher in PCOS compared to the non-PCOS controls (Table 2). A significantly shorter BF duration (months) was observed in patients with PCOS in comparison with non-PCOS controls (9.6 ± 7.0 vs. 12.6 ± 5.9, respectively; *P* < 0.001) and were less likely to practice exclusive BF at 6 months (39.6% vs. 62.9%, respectively; *P* < 0.001) (Table 3; Fig. 1).

**Table 3:** BF characteristics among patients with and without PCOS

Variable	PCOS (*n*. 101)	Non-PCOS (*n*. 140)	*P* value
BF duration (months)	9.6 ± 7.0	12.6 ± 5.9	**< 0.001**
Exclusive BF (n, %)	40 (39.6%)	88 (62.9%)	**< 0.001**
Partial BF (n, %)	28 (27.7%)	32 (22.8%)	0.452
FF (n, %)	33 (32.7%)	20 (14.3%)	**< 0.001**

Data are expressed as mean ± standard deviations for continuous variables and as number (percentage) for categorical variables. *P* values were calculated using Student’s t test, chi-square test, or Fisher’s exact test, as appropriate. BF: breastfeeding; FF: formula feeding; PCOS: polycystic ovary syndrome

Data were also stratified according to BF modality at 3 months postpartum (exclusive BF vs. partial BF/FF) in both women with PCOS and controls without PCOS. In women with PCOS, the prevalence of the composite cardiometabolic outcome was significantly lower in those who reported exclusive BF compared with those with partial BF/FF (36.6% [15/41] vs. 83.3% [15/18], respectively; OR 0.11, *P* = 0.0015). In controls without PCOS, a not statistically significant difference in the prevalence of the composite cardiometabolic outcome was observed in women who were exclusive BF group vs. women who were partial BF/FF (8.0% [4/50] vs. 16.7% [1/6], respectively; OR 0.43, *P* = 0.4453). When the analysis was repeated according to BF status at 6 months postpartum, the association remained directionally consistent in the PCOS subgroup, with a lower prevalence among women who were still BF compared with those who were not (41.7% [15/36] vs. 65.2% [15/23], respectively; OR 0.38, *P* = 0.1102). No differences were observed in controls (8.9% [4/45] vs. 9.1% [1/11], respectively for the two subgroups; OR 0.81; *P* = 1.000). Baseline demographic, clinical, and metabolic characteristics were comparable between groups, with no statistically significant differences observed (Supplementary Table S1).

Univariate analysis was performed to identify factors associated with the development of the composite cardiometabolic outcome after 18 months from delivery. Several clinical and BF-related variables significantly influenced the risk profile (Table 4). PCOS status was associated with a markedly increased risk of cardiometabolic impairment (OR 9.68, 95% CI 5.12–18.31; *P* < 0.001). Similarly, the history of PIH and higher pre-pregnancy BMI were significant predictors of adverse outcomes (*P* < 0.05 for both). Regarding infant feeding practices, BF duration demonstrated a significant protective effect, with the risk of cardiometabolic alterations decreasing for every additional month of lactation (OR 0.88, 95% CI 0.82–0.94; *P* < 0.001). Notably, compared to FF, exclusive BF was associated with a 90% reduction in the risk of reaching the composite endpoint (OR 0.10, 95% CI 0.04–0.25; *P* < 0.001).

**Table 4:** Univariate logistic regression analysis for the risk of composite cardiometabolic outcome at 18-month visit after delivery in high-risk patients with and without PCOS and history of PIH and/or GDM

Variable	OR	95% CI	*P* value
PCOS status (yes vs. no)	9.68	5.12–18.31	**< 0.001**
Maternal age (per year)	1.02	0.96–1.08	0.542
Pre-pregnancy BMI (per kg/m^2^)	1.15	1.07–1.24	**< 0.001**
Weight gain in pregnancy (per kg)	1.06	0.96–1.17	0.245
Weight loss after delivery (per kg)	0.96	0.89–1.04	0.312
PIH (yes vs. no)	3.42	1.95–6.01	**< 0.001**
GDM (yes vs. no)	1.48	0.85–2.58	0.165
BF duration (per month)	0.88	0.82–0.94	**< 0.001**
Exclusive BF vs. FF	0.10	0.04–0.25	**< 0.001**
Partial BF vs. FF	0.42	0.18–0.98	**0.046**
FF	1.00 (Ref)	—	—

BF: breastfeeding; BMI: body mass index; CI: confidence interval; FF: formula feeding; GDM: gestational diabetes mellitus; OR: odds ratio; PCOS: polycystic ovary syndrome; PIH: pregnancy induce hypertension

At multivariate logistic regression analysis, PCOS status and the history of PIH remained the strongest independent predictors of cardiometabolic risk after delivery (Table 5). However, even after adjustment, exclusive BF maintained a significant independent protective role (aOR 0.14, 95% CI 0.05–0.38; *P* < 0.001) (Table 5). When the multivariate model included pre-pregnancy metabolic profile (including PCOS status and pre-pregnancy BMI)exclusive BF remained a potent independent protector against the composite cardiometabolic outcome at 18 months from delivery (aOR 0.15, 95% CI 0.07–0.33; *P* < 0.001).

**Table 5:** Multivariate logistic regression analysis for independent predictors of long-term cardiometabolic impairment in high-risk patients with and without PCOS and history of PIH and/or GDM

Variable	aOR	95% CI	*P* value
PCOS status (yes vs. no)	7.12	3.45–14.68	**< 0.001**
PIH (yes vs. no)	2.85	1.42–5.72	**0.003**
GDM (yes vs. no)	1.24	0.62–2.48	0.540
Pre-pregnancy BMI	1.08	0.99–1.18	0.082
Exclusive BF	0.14	0.05–0.38	**< 0.001**

aOR: adjusted odds ratio; BF: breastfeeding; BMI: body mass index; CI: confidence interval; GDM: gestational diabetes mellitus; PCOS: polycystic ovary syndrome; PIH: pregnancy-induced hypertension

Stratified analysis by cohort origin (GDM vs. PIH/PE) confirmed the consistency of our findings. In the GDM cohort, exclusive BF was associated with a significant reduction in cardiometabolic risk (aOR 0.16, 95% CI 0.05–0.51), similar to the effect observed in the PIH/PE cohort (aOR 0.13, 95% CI 0.04–0.42). Formal interaction tests showed no significant interaction between the type of cohort and the protective effect of exclusive BF (*P* = 0.651), confirming the homogeneity of the pooled data.

## Discussion

To the best of our knowledge, this study represents the first longitudinal investigation specifically focused on the impact of BF in influencing the cardiometabolic risk profile of patients with PCOS who experienced pregnancy complications such as GDM and PIH.

These findings suggest a strong independent association between BF and reduced cardiometabolic risk in patients with PCOS and previous pregnancy complications, i.e. PIH and GDM. The multivariate logistic regression confirmed that both the duration of BF and the practice of exclusive BF are inversely associated with the risk of cardiometabolic impairment. Specifically, exclusive BF was associated with a 90% reduction in the risk of reaching the composite cardiometabolic endpoint, even after adjusting for maternal age, PCOS status, and pre-pregnancy BMI. These results further support the existence of an association between lactation and cardiometabolic outcomes. However, given the observational design of the study, a causal relationship cannot be established. It is possible that lactation is linked to a more favorable metabolic profile, potentially through mechanisms such as improved insulin sensitivity and vascular function, although alternative explanations, including reverse causation, cannot be excluded.

Our findings demonstrate that patients with PCOS have a significantly shorter BF duration of about 3 months and are less likely to practice exclusive BF at 6 months compared to non-PCOS controls. Earlier reports on BF in PCOS [18] also suggested that BF is impaired in women with PCOS. On the other hand, data from large community-based studies refused that hypothesis and found that obesity - and not PCOS per se - was associated with reduced BF initiation and duration rates [15, 17].

The specific mechanisms behind PCOS and lower BF rates remain unclear [16], even if both insulin resistance and hyperandrogenism in PCOS have been proposed as key factors inhibiting successful BF [16]. In this context, it is important to consider that the relationship between BF and metabolic health is likely bidirectional. On one hand, lactation has been associated with improved insulin sensitivity and metabolic regulation. On the other hand, underlying metabolic and hormonal disturbances typical of PCOS may impair lactogenesis. In particular, hyperandrogenism has been associated with breast hypoplasia and insufficient milk production, while insulin resistance may interfere with the hormonal pathways regulating milk synthesis [29]. Therefore, reduced BF may represent not only a contributing factor but also a consequence of an adverse metabolic profile. Accordingly, BF should be interpreted as a potential marker of metabolic health rather than a direct causal determinant. Thus, these hypotheses should be intended as speculative and not conclusive. In the current study the reduced lactation performance in the PCOS cohort was associated with a dramatically higher prevalence of cardiometabolic alterations at the 18-month follow-up, including a higher incidence of PH, DM2, and a worse overall metabolic profile characterized by elevated HOMA-IR, LDL cholesterol, and triglycerides. These findings further support an association between lactation and cardiometabolic outcomes. However, a causal relationship cannot be established, and reverse causation cannot be excluded.

A crucial consideration in interpreting these findings is the potential for reverse causation, as maternal metabolic health immediately following pregnancy may influence the ability to sustain lactation. It is biologically plausible that severe insulin resistance or hypertensive distress could interfere with lactogenesis [30], thereby predicting shorter BF duration rather than being a consequence of it. However, the sensitivity analysis conducted in this study, which adjusted for pre-pregnancy BMI and PCOS status, demonstrates that BF exerts a significant protective effect that is independent of the initial metabolic impairment. Unfortunately, other pre-pregnancy data were unknown in consideration of the study design and timing enrollment. While the relationship between maternal metabolism and lactation is likely bidirectional, our data supports the hypothesis that BF is linked to a more favorable cardiometabolic trajectory, suggesting its potential as a non-pharmacological intervention that mitigates the long-term cardiometabolic trajectory in high-risk patients with PCOS.

Obviously, current study has strengths and limitations. A major strength lies in its prospective cohort design and the use of standardized, contemporary diagnostic criteria for PCOS and metabolic conditions, including the most recent guidelines. However, certain limitations must be also considered. First, even if BF data were recorded longitudinally, they were re-analyzed in a retrospective fashion. We must also acknowledge the potential for recall bias, which is a common challenge in studies involving BF data. However, in our study, this risk was mitigated by the prospective nature of the original data collection, and by the use of structured and standardized interviews. Nevertheless, as a secondary analysis of existing data, we were limited to the information available in the original registries, and we cannot entirely rule out minor inaccuracies in maternal reporting. Second, it is important to consider the recruitment setting of our study. Participants were recruited from high-volume tertiary care hospitals, which inherently serves as a potential source of selection bias. Patients referred to these specialized units often present with a higher-risk profile, more severe PCOS phenotypes, or more significant unknown metabolic comorbidities than those managed in primary or secondary care settings. This higher baseline risk may limit the generalizability of our findings to the broader less-complicated pregnancies. The magnitude of BF benefit might differ in women with PCOS who experience uncomplicated pregnancies. Future community-based studies are needed to determine if these protective effects of lactation are equally pronounced across the full spectrum of PCOS severity. Third, we must acknowledge the potential for residual confounding regarding maternal lifestyle and socioeconomic status. Data on educational level, postpartum physical activity, and dietary adherence were not integrated into the final multivariable regression. This choice was dictated by the need to preserve model parsimony relative to our sample event size and to avoid over-adjustment, as lifestyle factors heavily mediate their metabolic effects through BMI changes - a covariate already adjusted for in our models. Nevertheless, the lack of direct adjustment for individual adherence to postpartum diet and exercise protocols remains a limitation, and we cannot entirely exclude that variations in these behaviors may have residually influenced both BF success and long-term cardiometabolic trajectories. Fourth, a distinct methodological limitation concerns the source data utilized for the cohort of women with and without PCOS who experienced PIH/PE [14]. The long-term follow-up data for this specific subpopulation are derived from a closely related extension study [14]. Because the final typeset proofs and a functional Digital Object Identifier (DOI) are not yet available at the time of this writing, the comprehensive, standalone peer-reviewed methodology of that primary study remains inaccessible to the public. Thus, readers should nevertheless interpret the secondary findings and conclusions derived from Cohort 2 with additional caution until its definitive publication. A potential concern in our study could also be the clinical heterogeneity arising from pooling patients with different pregnancy complications (GDM and PIH/PE) recruited from different centers. However, our stratified analysis and interaction tests demonstrated that the protective effect of BF remains robust and consistent regardless of the specific metabolic or vascular stressor experienced during pregnancy. This suggests that the physiological benefits of lactation on insulin sensitivity and vascular health are universally applicable to high-risk PCOS populations, further supporting the generalizability of our conclusions. Finally, we must acknowledge the limitations inherent in the secondary nature of this analysis and the available sample size. While the study was adequately powered for the primary composite outcome, the number of events in certain sub-categories—specifically type 2 diabetes mellitus (DM2)—was low. This sparsity of data for individual components of the cardiometabolic profile may increase the risk of effect size overestimation for these specific conditions. In the PCOS subgroup, the marked difference observed between BF groups (36.6% vs. 83.3%) corresponded to a large effect size (Cohen’s h = 1.00) and a high post-hoc statistical power (94.3%), supporting the robustness of the primary composite outcome analysis and reinforcing the strength of the observed association. However, given the limited number of events for individual outcomes, these sub-analyses should be interpreted with caution and considered exploratory and hypothesis-generating rather than definitive.

In conclusion, BF is significantly associated with a lower prevalence of long-term persistence of hypertension and glucose metabolism abnormalities in women with PCOS who have a history of GDM and/or PIH. These findings suggest that BF may represent a potentially relevant marker and modifiable factor associated with cardiometabolic risk in this high-risk population, although causal relationships cannot be established. Supporting and optimizing lactation in women with PCOS may therefore be clinically relevant, particularly among those at very high risk. Future research should prioritize larger, multicenter prospective studies to investigate the biological mechanisms underlying these protective effects, such as the potential improvement in insulin sensitivity, and to evaluate whether these benefits vary across different PCOS phenotypes.

## Supplementary Information

Below is the link to the electronic supplementary material.

Supplementary Material 1

## Data Availability

Data available on reasonable request to the corresponding author.
