# Supplementary material for: Effect of breastfeeding on cardiometabolic risk in patients with polycystic ovary syndrome
Source: J Endocrinol Invest. 2026 Jun 22;49(10):2845–54. doi: 10.1007/s40618-026-02948-7 (PMC13627247; doi:10.1007/s40618-026-02948-7)
Supplement: Supplementary file 1 [file 40618_2026_2948_MOESM1_ESM.docx]

**Supplementary Table S1.** Baseline demographic and obstetric characteristics of women with PCOS according to BF modality.

| Variable | Exclusive BF | Partial/FF | *P* value |
| --- | --- | --- | --- |
| Age, years | 30.3 ± 5.9 | 30.8 ± 4.7 | 0.7635 |
| Weight, kg | 69.1 ± 10.5 | 73.7 ± 14.1 | 0.2236 |
| Height, cm | 159.9 ± 8.6 | 163.7 ± 6.8 | 0.0742 |
| BMI, kg/m² | 27.2 ± 5.0 | 27.7 ± 6.0 | 0.7970 |
| Spontaneous conception | 24/41 (58.5%) | 10/18 (55.6%) | 0.1458 |
| Ovulation induction | 8/41 (19.5%) | 7/18 (38.9%) |  |
| IVF | 9/41 (22.0%) | 1/18 (5.6%) |  |
| Multiparous | 17/41 (41.5%) | 4/18 (22.2%) | 0.2383 |
| Gestational age at diagnosis, weeks | 28.1 ± 4.7 | 30.1 ± 3.7 | 0.0887 |
| Birth weight, g | 3670.5 ± 748.2 | 3638.9 ± 737.4 | 0.8810 |
| Gestational weight gain, kg | 13.6 ± 5.4 | 13.7 ± 5.8 | 0.9599 |

Data are presented as mean ± SD or n/N (%), as appropriate. *P* values were calculated using Student’s t test, chi-square test, or Fisher’s exact test, as appropriate. BF, breastfeeding; FF, formula feeding; IVF, in vitro fertilization
